# Interaction of iron phthalocyanine with the graphene/Ni(111) system

**DOI:** 10.3762/bjnano.5.34

**Published:** 2014-03-17

**Authors:** Lorenzo Massimi, Simone Lisi, Daniela Pacilè, Carlo Mariani, Maria Grazia Betti

**Affiliations:** 1 Dipartimento di Fisica, Università di Roma La Sapienza, Piazzale A. Moro 2, I-00185 Roma, Italia; 2 Dipartimento di Fisica, Università della Calabria, I-87036 Arcavacata di Rende (CS), Italia

**Keywords:** angular-resolved photo-electron spectroscopy (ARPES), buffer layer, graphene, molecule–substrate interaction

## Abstract

Graphene grown on crystalline metal surfaces is a good candidate to act as a buffer layer between the metal and organic molecules that are deposited on top, because it offers the possibility to control the interaction between the substrate and the molecules. High-resolution angular-resolved ultraviolet photo electron spectroscopy (ARPES) is used to determine the interaction states of iron phthalocyanine molecules that are adsorbed onto graphene on Ni(111). The iron phthalocyanine deposition induces a quenching of the Ni d surface minority band and the appearance of an interface state on graphene/Ni(111). The results have been compared to the deposition of iron phthalocyanine on graphene/Ir(111), for which a higher decoupling of the organic molecule from the underlying metal is exerted by the graphene buffer layer.

## Introduction

The interest in the preparation of ordered layers of organic molecules is rapidly growing, because of the possibility to realize advanced electronic- and spin-based devices [1–3]. Transition-metal phthalocyanines (MPcs) are planar molecules that consist of an organic cage formed by four pyrrole and benzene rings with a central metal ion [4]. They represent a class of molecules with potentially large applications thanks to their easily tunable electronic and magnetic properties, which are basically associated with the electronic configuration of the central metal atom [5]. When deposited on surfaces, their interaction may be driven by dipolar forces mainly related to the organic cage and by a stronger interaction that is associated with the central metal atom. As an example, the adhesion of iron phthalocyanine (FePc) and cobalt phthalocyanine (CoPc) on a Au substrate is mainly due to the presence of unfilled, out-of-plane oriented, d states that interact with the underlying gold states [6–7].

The magnetic and electronic properties of the adsorbed molecules may be strongly influenced by the interface and can be potentially tuned by using an appropriate buffer layer. Graphene (Gr), thanks to its unique electronic properties [8] and to the quite easy experimental preparation on many metal substrates [9], is a good candidate to tune the MPc–metal interface. Moreover, the self-assembling capabilities of organometallic molecules offer the possibility to form ordered networks of metal atoms trapped in an organic cage, which is a suitable configuration for the realization of spin-based qubits [10]. Interesting and exemplary cases are represented by MPcs adsorbed on graphene grown on Ni(111) and Ir(111) surfaces. In fact, graphene on Ni(111) and on Ir(111) represents two opposite sides of the graphene–metal interaction: a strong interaction with a strong modification of the free-standing graphene band structure is observed on Ni [11], while a low interaction with an almost unperturbed Dirac cone is present if graphene is grown on Ir [12–13]. Recently it has been shown that graphene acts as a buffer layer that decouples the FePc molecules from Ir(111) and prevents an Ir–FePc interaction [13]. On the other hand, for Gr/Ni(111) a FePc–Ni interaction has been suggested [14–16] despite the presence of the graphene sheet, as it was already observed for CoPc on Gr/Ni(111) [17]. We present a valence band UV photoemission study of the FePc adsorption on Gr/Ni, which brings to light a direct evidence of an interaction between the FePc molecule and the Ni substrate.

## Experimental

Experiments were performed in situ in ultra-high-vacuum (UHV) chambers with base pressures in the low 10^−10^ mbar range at the LOTUS laboratory of the Università La Sapienza (Roma). The Ni(111) single crystal was cleaned by several sputtering–annealing cycles (1 keV Ar^+^ for 30 min, 600 °C for 10 min). Graphene was obtained by exposing the sample, which was kept slightly below 600 °C to 6000 L of ethylene (1 L = 10^−6^ torr·s). The formation of graphene on the Ni(111) surface is complicated by the segregation of carbon from the bulk, because of the high solubility of carbon in Ni [18–20]. The Ir(111) single crystal was cleaned by several sputtering–annealing cycles (2 keV Ar^+^ for 30 min, 1200 °C for 60 s). The preparation of graphene was done by several 120-seconds long exposures to ethylene while flash-heating the sample up to 1100 °C.

The deposition of FePc was carried out by using a custom-made quartz crucible and the molecular deposition was controlled by using a quartz microbalance. One single-layer (SL) is defined as the molecular density of flat molecules fully covering the graphene layer, and it corresponds to a nominal thickness of about 3.4 Å.

Low energy electron diffraction (LEED) was used to check the symmetry of both clean and Gr-covered surfaces. LEED was performed in the energy range of the primary beam of 90–140 eV. High-resolution angular-resolved photoelectron spectroscopy data was carried out by using a SCIENTA SES-200 analyzer with an angular acceptance of ±8° and a resolution of 16 meV. All spectra have been taken along the ΓK direction. The UV radiation, HeI*α* (21.218 eV) and HeII*α* (40.814 eV), was provided by a SCIENTA VUV-5050 monochromatic source.

## Results and Discussion

**Figure 1:**
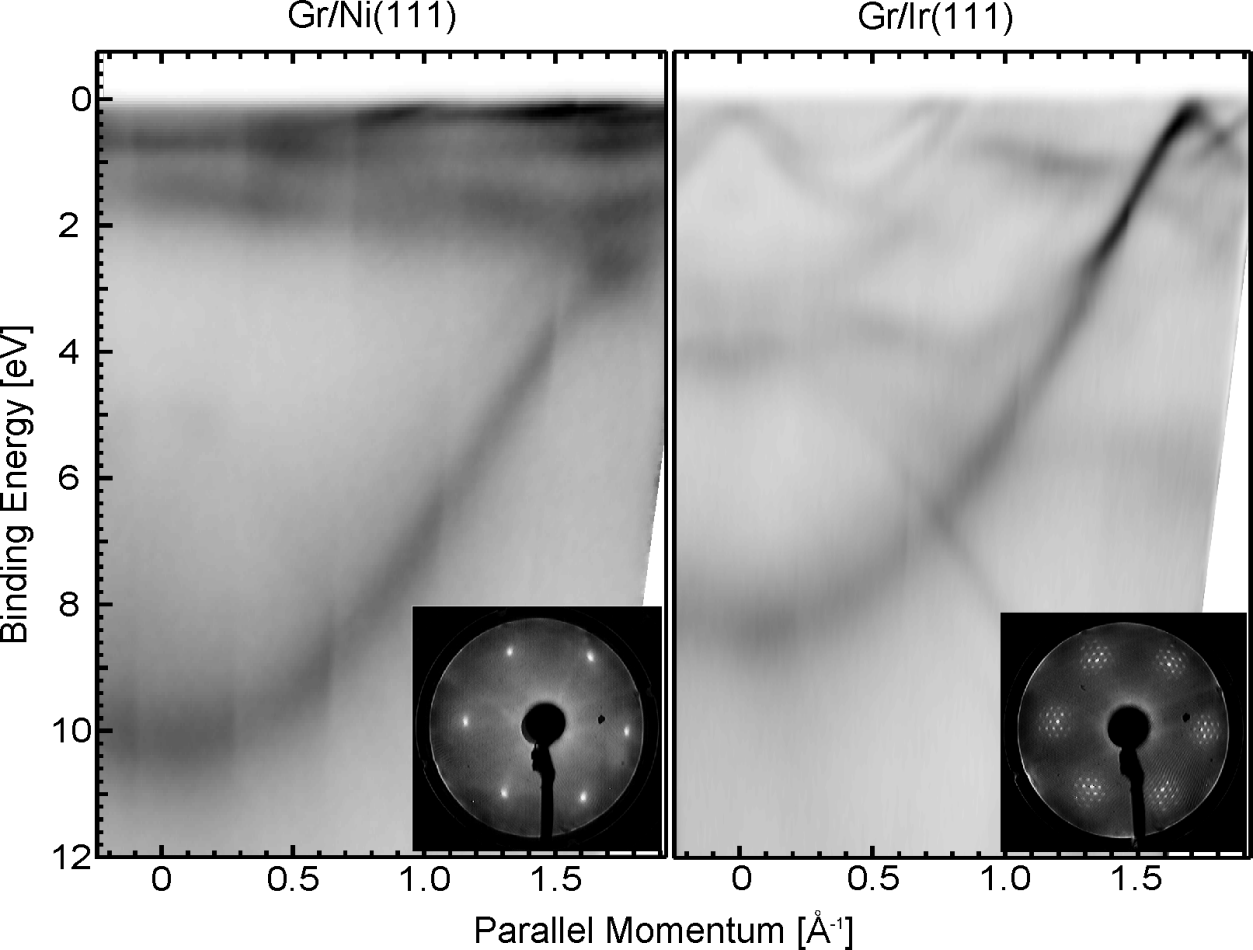
Experimental ARPES band structure for graphene grown on Ni(111) (left) and on Ir(111) (right), taken with 40.814 eV photon energy along the ΓK direction of the 2D BZ. Insets: corresponding LEED patterns taken on Gr/Ni and Gr/Ir, at primary beam energies of 90 eV and 140 eV, respectively. LEED patterns have been obtained by using a different geometry.

The ARPES band structure of the Gr/Ni(111) and Gr/Ir(111) systems along the ΓK direction of the two-dimensional (2D) Brillouin Zone (BZ) is presented in Figure 1. The corresponding LEED patterns are shown in the insets: for Gr/Ni(111) the graphene lattice is well aligned with the substrate and no corrugation is present, with a resulting (1 × 1) symmetry [21–22], while in Gr/Ir(111) the lattice mismatch, reflected in the additional moiré pattern, introduces a large-scale regular corrugation [23].

The presence of a distinct single π band for both Gr sheets (on Ni and on Ir) reveals the single-layer nature of graphene. For Gr/Ir(111), the Dirac point is localized on a projected bulk band gap and the graphene π band looks very similar to the band of free-standing graphene, the linear dispersion of the π band is preserved close to the K point with only a slight p-doping, which is in agreement with the literature [12–13]. The very tiny doping has also been interpreted as slight hybridization between the Gr-π states and the underlying Ir d bands, which leads to a gap with a width of a few tens of meV [24]. The small size of the gap can be explained by the small difference among the two sublattices, due to the low interaction with the substrate. The band structure of the Gr/Ni(111) system appears to be dominated by the strong projected Ni d bands very close to the Fermi level. Furthermore, the Gr-π band is shifted by 2.5 eV towards higher binding energies as compared to Gr/Ir, and no linear dispersion is observed at the K point, which is in agreement with previous results [11]. Carbon atoms adsorb on two different sites on Ni(111), on top of Ni surface atoms and in fcc-hollow sites of the underlying Ni mesh [21,25]. As a consequence of the different adsorption sites and of the strong interaction, a large asymmetry among the two carbon sublattices is introduced, which induces a band-gap opening [11]. Recent experiments performed along the BZ direction perpendicular to ΓK confirm the strong shift of the Dirac point, while the gap opening is attributed to a strong hybridization of the Gr-π^*^ states with the Ni d bands [22].

**Figure 2:**
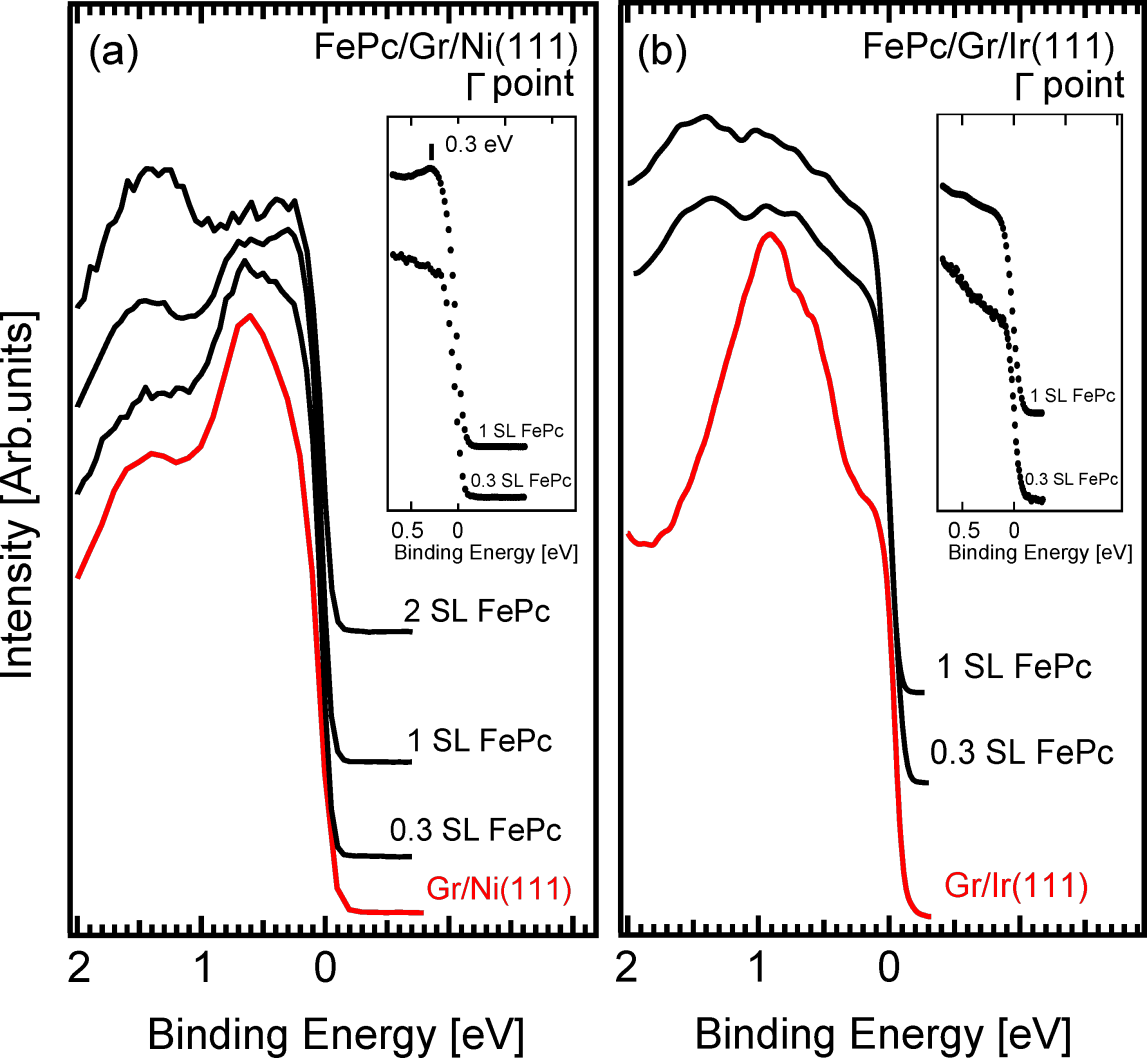
Valence band photoemission data for the adsorption of FePc onto Gr/Ni (a) and onto Gr/Ir (b), as a function of the thickness of FePc layer. Data of clean graphene (red lines) and of the FePc/Gr systems (black lines). Data taken with 40.814 eV photon energy (HeII*α*) and around normal emission (±4° angular integration around the Γ point). The data was normalized to the intensity at the Fermi edge and vertically stacked for clarity. In the insets, a zoom around the Fermi level for a coverage of 0.3 and 1 SL of FePc is given.

The photoemission data in the low binding energy region for the iron phthalocyanine molecules deposited on the Gr/Ni surface, and on Gr/Ir for comparison, as a function of the thickness of the FePc layer is shown in Figure 2. The FePc adsorption on Gr/Ni(111) produces a general reduction of the prevalent d band spectral density of states and a new feature emerges close to the Fermi level (at about 0.3 eV BE). Its intensity grows upon increasing the molecular coverage up to completion of the first SL, and starts to decrease at higher thicknesses, a behaviour typical of an interface state. This interface state comes from charge transfer from the Gr/Ni substrate to partially filled d orbitals of the central metal ion [15]. On the other hand, the deposition of FePc on the Gr/Ir surface causes only a general attenuation of the Gr/Ir spectral density, while no new state emerges, which confirms previous results [13]. The presence of an interface state for FePc adsorbed on Gr/Ni but not for FePc adsorbed on Gr/Ir can be very likely related to an interaction of the FePc molecules with the substrate underlying the graphene layer. In fact, the shorter distance of Gr–Ni compared to Gr–Ir [25–26] may induce a larger overlapping of the partially empty out-of-plane d like orbitals of FePc with the hybridized d–π states of Ni–Gr.

**Figure 3:**
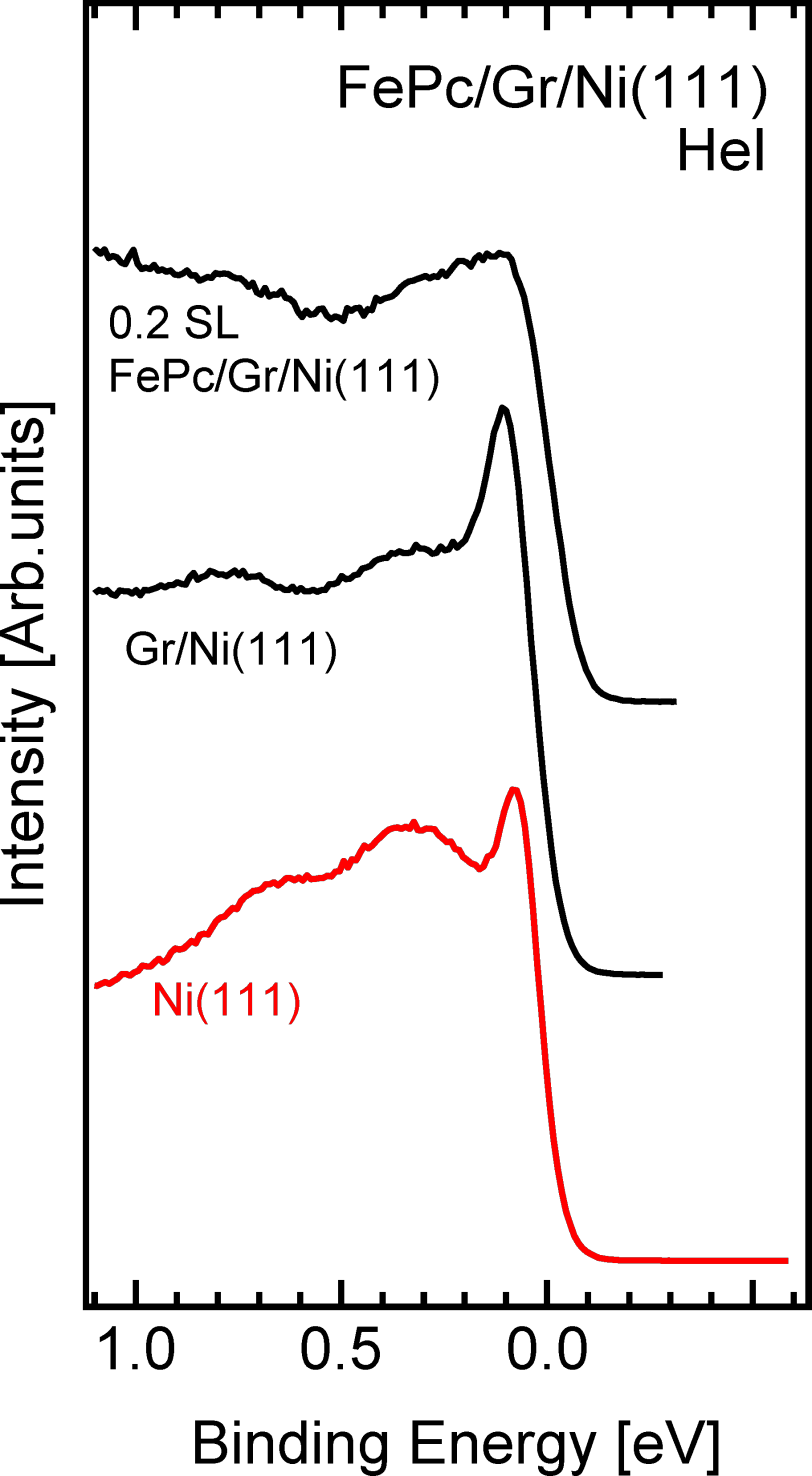
Valence band spectral density of states of clean Ni(111) (red line), of Gr/Ni(111) and of 0.2 SL FePc onto Gr/Ni (black lines), taken at the K point of the BZ (±2° angular integration around K, with 21.218 eV photon energy).

In order to better understand the nature of the interaction, we also analyze data at the K point of the BZ, as shown in Figure 3. At the clean Ni(111) surface (Figure 3, bottom spectrum), we observe two main peaks close to the Fermi level, at 0.08 eV and 0.30 eV BE, respectively. As it is well known, the first one is attributed to d electron minority spin with sp-contribution and the second one to the majority spin [27]. The formation of graphene onto Ni(111) induces an increase in intensity accompanied by a very slight shift (−0.02 eV) of the first feature, while the d majority band at 0.3 eV BE remains unchanged and shows only a slight intensity reduction. The observed change of the minority d band originates from a hybridization with the graphene π bands [25]. The increased intensity of the lowest BE peak is emphasized by the high excitation cross-section for the C π-bands with respect to the Ni d-like states [28], which brings to light the hybrid nature of this first peak. Upon adsorption of a tiny quantity of FePc, this hybrid state is strongly reduced in intensity, while the d majority band appears to be basically unaffected. The strong reduction in intensity of the π–d hybrid state suggests a molecule interaction with the Gr/Ni(111) interface, which validates the suggestion of a molecule–substrate interaction that is mediated by graphene. This is in agreement with recent investigations, in which electron energy loss and photoemission spectroscopy were used [14–15].

## Conclusion

When used as a buffer layer between an organic molecule and a metal surface, graphene plays a different role in the molecule–metal interaction that depends on the interaction of graphene with the metal substrate. Graphene on Ni(111) reveals a strong interaction with the substrate and strong alteration of the ideal graphene π band. After deposition of small amounts of FePc molecules, by means of high-resolution UV photoemission we give direct experimental evidence of an interaction of the molecule with Ni through graphene, as shown by the emerging of an interface state at about 0.3 eV binding energy in normal emission and by the quenching of the Gr–Ni π−d hybrid state at the K point of the BZ.
